# Evaluating environmental impacts of selection for residual feed intake in pigs

**DOI:** 10.1017/S175173112000138X

**Published:** 2020-12

**Authors:** T. Soleimani, H. Gilbert

**Affiliations:** GenPhySE, Université de Toulouse, INRAE, F-31326, Castanet-Tolosan, France

**Keywords:** feed efficiency, life cycle assessment, growth performance traits, selection by genetics, net energy flux

## Abstract

To identify a proper strategy for future feed-efficient pig farming, it is required to evaluate the ongoing selection scenarios. Tools are lacking for the evaluation of pig selection scenarios in terms of environmental impacts to provide selection guidelines for a more sustainable pig production. Selection on residual feed intake (**RFI**) has been proposed to improve feed efficiency and potentially reduce the associated environmental impacts. The aim of this study was thus to develop a model to account for individual animal performance in life cycle assessment (**LCA**) methods to quantify the responses to selection. Experimental data were collected from the fifth generation of pig lines divergently selected for RFI (low line, more efficient pigs, **LRFI**; high line, less efficient pigs, **HRFI**). The average feed conversion ratio (**FCR**) and daily feed intake of LRFI pigs were 7% lower than the average of HRFI pigs (*P* < 0.0001). A parametric model was developed for LCA based on the dietary net energy fluxes in a pig system. A nutritional pig growth tool, InraPorc^®^, was included as a module in the model to embed flexibility for changes in feed composition, animal performance traits and housing conditions and to simulate individual pig performance. The comparative individual-based LCA showed that LRFI had an average of 7% lower environmental impacts per kilogram live pig at farm gate compared to HRFI (*P* < 0.0001) on climate change, acidification potential, freshwater eutrophication potential, land occupation and water depletion. High correlations between FCR and all environmental impact categories (>0.95) confirmed the importance of improvement in feed efficiency to reduce environmental impacts. Significant line differences in all impact categories and moderate correlations with impacts (>0.51) revealed that RFI is an effective measure to select for improved environmental impacts, despite lower correlations compared to FCR. Altogether more optimal criteria for efficient environment-friendly selection can then be expected through restructuring the selection indexes from an environmental point of view.

## Implications

Selection on feed efficiency results in large correlated reductions in the environmental impacts of pig production; with gross feed efficiency having more impact than net feed efficiency. Our pig-based evaluation model will allow definition of selection criteria that result in even larger reductions in environmental impact.

## Introduction

Beyond being an economic bottleneck, feed greatly contributes to the environmental impacts of pig farming (McAuliffe *et al.*, 2016). Improvement in feed efficiency is a major goal for pig production sustainability, because it reduces environmental fluxes associated with feed production (Nguyen *et al.*, 2011) and reduces the amount of effluent per pig as a result of mass balance (Ali *et al.*, 2018). Feed efficiency, which is usually inversely expressed as feed conversion ratio (**FCR**), stands for the BW gain per unit of feed consumed. Selection for FCR, directly or via increased growth rate or reduced fatness, has been very effective to improve feed efficiency in the past. However, as a ratio, FCR is closely correlated with production traits, and selection on this trait has uncontrolled effects on the components of the ratio (Saintilan *et al.*, 2013). In 1963, Koch *et al.* introduced a more targeted indicator for net feed efficiency, residual feed intake (**RFI**). The RFI, which is a linear combination of traits, is moderately heritable in pigs (Saintilan *et al.*, 2013) and is defined as the difference between observed feed intake and the feed intake expected from individual maintenance and production requirements. Among the range of approaches for measuring feed efficiency, RFI is increasingly becoming the measure of choice in some species (Kenny *et al.*, 2018). Improving animal feed efficiency is possible at two stages. The first stage, which arises from the interaction between feed and animal in the digestive tract, is to improve conversion of the feed gross energy into metabolisable energy (ME). The second stage is to improve the partitioning of uptaken energy between maintenance and tissue accretion through protein (**PD**) and lipid deposition (**LD**) (Nguyen *et al.*, 2005). Improving feed efficiency through selection based on RFI essentially corresponds to the latter (Gilbert *et al.* 2017). Separate selection for RFI has been investigated and impacts on production performance (Gilbert *et al.*, 2007; Cai *et al.*, 2008) as well as on sow reproduction and piglet traits were reported (Gilbert *et al.*, 2012; Young *et al.*, 2016). However, to date, its impacts have not been thoroughly assessed from an environmental viewpoint due to the lack of an appropriate model. To quantify environmental impacts, several studies using life cycle assessment (**LCA**) examined the environmental burdens of different pig production options (Garcia-Launay *et al.*, 2014; Mackenzie *et al.*, 2015; McAuliffe *et al.*, 2017). The aim of the present study was to develop a model adapted to the evaluation of pig selection strategies and use it to estimate the environmental impacts of selection for RFI, through comparative LCA of two lines of pigs divergently selected for RFI.

## Material and methods

### Experimental data

The experimental data were obtained from the fifth generation of Large White pigs divergently selected for RFI. The selection process and results concerning low RFI (LRFI, more efficient pigs) and high RFI (HRFI, less efficient pigs) lines are reviewed in Gilbert *et al.* (2017). The present data set includes 60 male pigs in the LRFI line and 58 male pigs in the HRFI line. Growing pigs had *ad libitum* access to a one-phase conventional diet (Table 1). The experimental data were collected from birth to slaughter. Body weight was recorded at birth; at weaning (average 28 days of age); at the beginning of the fattening period (10 weeks of age); at 11, 15, 19, and 23 weeks of age; and at the end of the test (target BW 115 kg). During the fattening period, data on individual daily feed intake (**DFI**) recorded on ACEMA 64 automatic feeders (ACEMO, Pontivy, France) were available, and back fat thickness (**BFT**) was measured by ultrasound on live animals at 23 weeks of age, using an ALOKA SSD-500 echograph (Aloka, Cergy Pontoise, France). From these records, FCR and RFI were computed as described in Gilbert *et al.* (2007). For LRFI and HRFI sows/litters, the mean values of age at farrowing and weaning, sow BW and BFT before farrowing and at weaning, lactation DFI, number of total born, stillborn, weaned piglets, piglet BW at birth and at weaning and weaning age were taken from the experimental data presented in Gilbert *et al.* (2012).

**Table 1:** Ingredients, chemical composition and nutritional value of the experimental diet of pig lines

Item	Quantity
Ingredient (g/kg)	
Barley	409
Soft wheat	327
Soybean meal (48% CP)	202
Sunflower oil	23
l-Lysine HCL	3.5
l-Threonine	1.4
l-Tryptophane	0.3
dl-Methionine	0.9
Salt	4.5
Calcium carbonate	11
Dicalcium phosphate	12
Oligo vitamins	5
Chemical composition (g/kg)	
Ash	58.5
Dry matter	877.7
Organic matter	819.2
CP	172.3
Starch	411.9
Gross energy (MJ/kg)	16.22
NDF	141.7
ADF	47.4
Crude fibre	38.1
Residue	163
Calcium	9.97
Phosphorus	6.21
Nutritional value	
NE^1^ (MJ/kg)	9.70
ME^1^ (MJ/kg)	13.09
Std.dig. Lysine^2^ (g/kg)	9.83

NE = net energy; ME = metabolisable energy.

1 Calculated according to the method of Sauvant *et al.* (2004).

2 Standardised ileal digestible lysine.

### Goal, scope and framework of the environmental assessment

**Figure 1:**
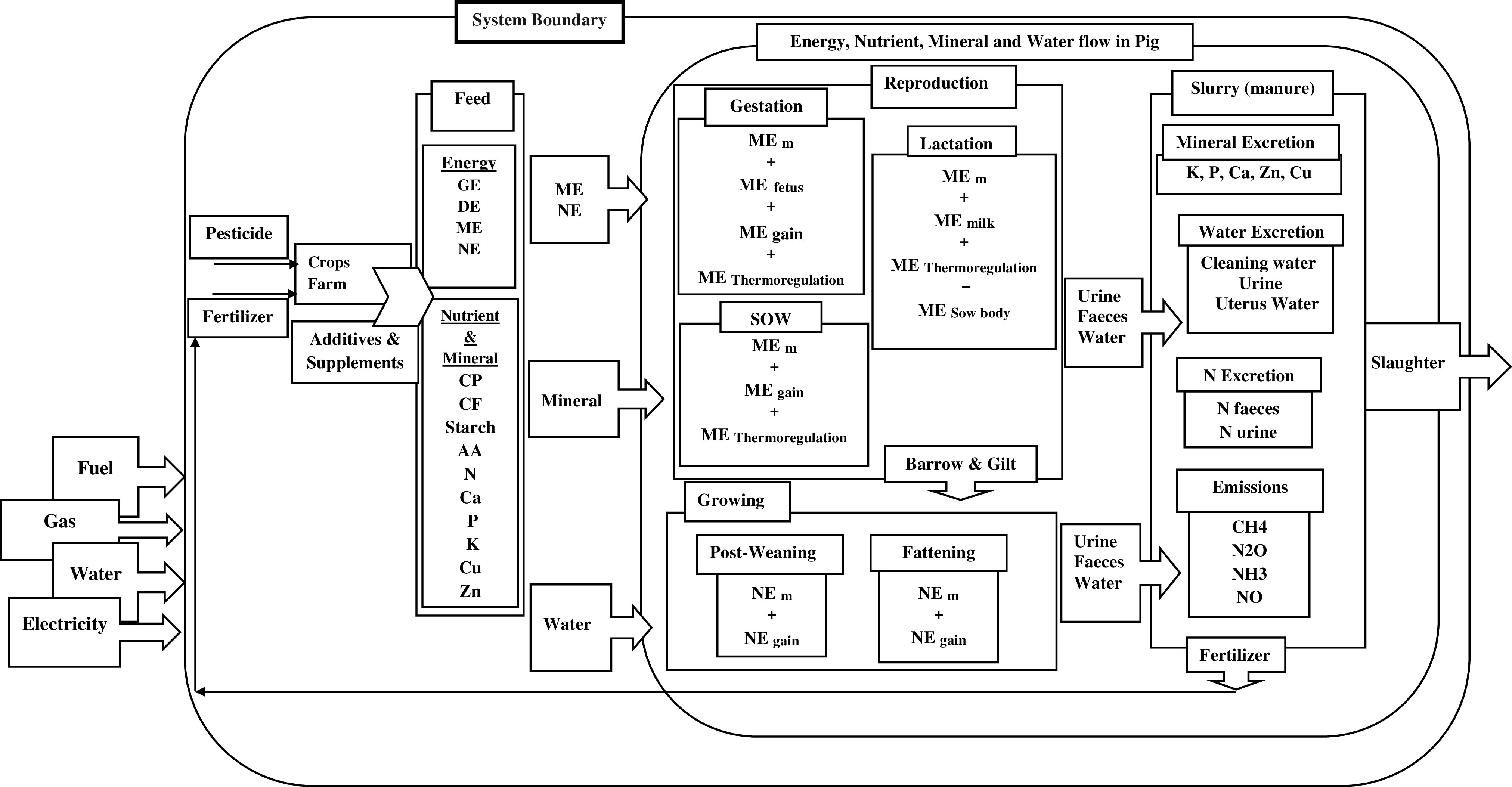
Scheme of the system boundary, which includes the entire pig farm, feed production processes and manure management. GE = gross energy; DE = digestible energy; ME = metabolisable energy; NE = net energy; ME_m_ = metabolisable energy required for maintenance; NE_m_ = net energy required for maintenance; NE _gain_ = net energy required for gain; CF = crude fibre; AA = amino acid; N = nitrogen; Ca = calcium; P = phosphorus; K = potassium; Cu = copper; Zn = zinc.

A ‘cradle-to-farm-gate’ system boundary was chosen, including feed production, manure management and the entire pig production system comprising reproducing sows and their piglets, post-weaning and fattening pigs. One kilogram of live weight (**LW**) of pig at the farm gate was used as the functional unit with the goal of comparing the environmental impacts between the HRFI and LRFI lines. To implement LCA, all the materials and energy consumed in the production of one functional unit of the system have to be included in the life cycle inventory (**LCI**), in addition to all excretions and emissions to the environment. The LCI needs to consider all the processes that take place inside the system boundary. To obtain a flexible and predictive model for daily feed intake, it was required to switch from the mass context of the data recording to the energy context for modelling. Due to the pigs’ ability to adapt their feed intake to the net energy (**NE**) concentration of different diets (Quiniou and Noblet, 2012), the model was developed based on the daily NE supply during fattening to allow prediction for different diet compositions and guaranty generality. Our model was consequently developed based on NE for the fattening period and ME for reproducing sows, to estimate the flux of dietary energy which propagates through all individual pigs within the system boundary (Figure 1).

### Model structure

The model consists of six modules with distinct functions.

#### Feeding plan module

InraPorc^®^, which is a model and software designed to simulate the performance response of pigs to different nutritional strategies (Dourmad *et al.*, 2008; van Milgen *et al.*, 2008), was incorporated in the LCA model to benefit from its features. It contains the licensed INRA-AFZ database of characterised feed ingredients (Sauvant *et al.*, 2004) as an embedded library. This library distinguishes different nutritional values depending on the animal physiological status (sows and growing pigs). In the feeding sub-module, the composition of the diet and the feeding plan (rationing and sequencing plan) during the different periods of the animal’s lifetime were defined based on experimental data. The outcome of this sub-module is the chemical compositions and nutritional values of the diets, based on the INRA-AFZ database.

#### Animal profile module

Each animal profile is the compilation of the feeding plan, housing conditions, experimental data, NE system and a final calibration in InraPorc^®^. The Gamma function was used to express *ad libitum* feed intake because of its flexibility which enables it to adjust to changes in feed intake and BW (van Milgen *et al.*, 2008). The daily *ad libitum* feed intake and NE of the feed characterised the animal daily NE requirements. InraPorc^®^ was used to establish the individual profiles for each pig separately in the lines during the fattening period (day 68 to day 179), based on the animal’s individual data, which were recorded daily, as previously proposed by Saintilan *et al.*, 2015. The average profiles for groups of sows and their piglets were defined separately in InraPorc^®^ based on the experimental data on the average HRFI and LRFI sows/litters performance summarised by Gilbert *et al.* (2012). The outcome of this module is the predicted growth performance (average daily gain (**ADG**) and average daily feed intake (**ADFI**)), PD and LD during fattening, respectively, the ratio of body protein to and body lipid (**BP**/**BL** ratio) and mineral excretions of the pigs. As InraPorc^®^ was not designed to model the performance of animals post-weaning, a calculation module was developed in R to estimate the excretions and emissions during the post-weaning period (28 days to 10 weeks of age), according to Rigolot *et al.* (2010a and 2010b).

#### Emission and excretion module

To calculate the emissions and excretions, and the slurry composition, three sub-modules were developed in R for the sow-litter, post-weaning and fattening stages. The average performance data were used for the sow-litter stage and the individual performance data were used for the post-weaning and fattening stages. The components of the excreta (DM, organic matter (**OM**), nitrogen (**N**), phosphorus (**P**), potassium (**K**), copper (**Cu**) and zinc (Zn) were calculated using the mass-balance approach, as the difference between nutrients taken up from the feed and the nutrients retained in the body. Emissions of enteric methane (**CH**_**4**_), nitrogen monoxide (**NO**), nitrous oxide (**N**_**2**_ **O**), ammonia (**NH**_**3**_) and carbon dioxide (**CO**_**2**_) during housing were calculated according to Rigolot *et al.* (2010a and 2010b). Subtraction of N excretion and gaseous N lost in housing determined the quantity of N at the beginning of manure storage (Garcia-Launay *et al.*, 2014).

A sub-module was developed to estimate emissions, leaching and runoff during manure storage and application in the field. The NH_3_ emissions during outside storage were calculated according to the emission factors recommended by Rigolot *et al.* (2010b). The NO_x_ emissions were calculated according to Nemecek *et al.* (2004). Methane emissions from manure during storage were calculated using guidelines by the Intergovernmental Panel on Climate Change (IPCC, 2006). Direct and indirect emissions of N_2_O and NH_3_ during the spreading of slurry were calculated according to IPCC (2006). The value of the manure as a replacement for synthetic fertiliser was considered according to the mineral fertiliser equivalency of 75% for N (Nguyen *et al.*, 2010) and 100% for P and K (Nguyen *et al.*, 2011).

#### Water, energy expenditure and transport modules

The model linked drinking water to feed intake according to the Institut de la Filière porcine (**IFIP**) report on typical French farms (IFIP, 2014), with water to feed ratios of 4.5, 4.0, 2.5 and 2.7 for lactating, gestating, post-weaning and fattening pigs, respectively. Cleaning water was estimated at 2300 l per sow and 30 l per fattening pig according to IFIP (2014) and Rigolot *et al.* (2010a). In addition, the energy expenditure link to the functional unit was 0.42 kWh/kg LW and was broken down into electricity, oil and gas components, according to IFIP (2014). Transport of feed was calculated as a coefficient of feed intake. Linking water and transport to feed intake made the model sensitive to feed efficiency for further sensitivity and uncertainty analyses.

### Life cycle impact assessment

An individual LCA was conducted for each pig in the LRFI and HRFI lines through incorporating its own experimental recorded traits and the traits obtained from InraPorc^®^ in the LCA model. The outputs of the LCA model were the impact categories of climate change (**CC**), terrestrial acidification potential (**AP**), freshwater eutrophication potential (**EP**), land occupation (**LO**) and water depletion (**WD**). For impact analyses, the ReCiPe Midpoint 2016 (H) V1.13 (Huijbregts *et al.*, 2016), one of the most recently updated life cycle impact assessment methods, with the Ecoalim (Wilfart *et al.*, 2016) and Ecoinvent (Wernet *et al.*, 2016) inventory databases, were used. The equivalency factors for the impact categories were assigned according to the factors recommended in the ReCiPe method. All environmental impact assessments were implemented in the SimaPro V8.5.4.0 on the MEANS (MulticritEria AssessmeNt of Sustainability) platform (http://www.inra.fr/means).

The line impact differences were tested with a *t* test, and impacts were declared significantly different for *P* < 0.05. In addition, correlations between performances and environmental impacts were calculated within lines, for a better understanding of the relationships between the components.

### Uncertainty analysis

Monte Carlo simulations is an approach, available in SimaPro V8.5.4.0, to quantify the effects of the uncertainties in the model parameters on the estimated environmental impacts: by resampling the parameter values based on assumptions about their uncertainties, a confidence interval for each impact can be obtained. In addition, the Ecoinvent LCA databases, which are embedded in SimaPro V8.5.4.0, provide quantitative uncertainties for parameters in most of its processes, mainly with log normal distributions (Ivanov *et al.*, 2019). To incorporate the intended traits in the LCA, a trait-based model was developed based on the growth performance equations presented by van Milgen *et al.* (2008) (also applied in InraPorc^®^) and linked to the emissions and excretions according to Rigolot *et al.* (2010a and 2010b). The quantities of all feed ingredients were linked to the related traits, such as ADFI and fattening duration, by considering their incorporation rate in the diet. This integrated and connected model made it possible to perform uncertainty and sensitivity analyses in SimaPro. To evaluate the impact of the LCA model parameter uncertainty on the results, the line mean values of the performance traits (ADFI, FCR, ADG, BP/BL ratio, PD, fattening duration, BP and BL at slaughter and BFT) were extracted from the experimental data and InraPorc^®^ outputs and used as inputs for the uncertainty analysis. Then the parallel Monte Carlo simulations were run on the two lines jointly to evaluate the sensitivity of the impact categories to the model parameter uncertainties.

### Sensitivity analysis

Sensitivity analysis is the study of the relative importance of the different input parameters in the model outputs. To perform a sensitivity analysis, it is necessary to have a parametric model in which all the parameters are mathematically interlinked (Supplementary material S1). To perform the sensitivity analysis on animal performance traits, related traits had to be incorporated in the model as direct input parameters accompanied by their distributions. In this way, any change in animal traits propagates through the model and affects the appropriate material, process, emission and excretion sub-inventories in the LCI.

An one-at-a-time (**OAT**) sensitivity analysis, an appropriate approach for limited parameter and linear LCA models, was conducted based on the upper and lower bounds of the 95% confidence interval (CI) (±2 SD) of the main production trait distributions. The LCA model was considered sensitive to a trait if a change in any impact value was greater than 5% after a change to the upper and lower bounds of the intended trait compared to the initial impact value (Mackenzie *et al.*, 2015). The OAT sensitivity analysis of the traits made it possible to identify the best candidate traits for improvement in the corresponding environmental impact categories.

## Results

### Traits comparison between lines

Prior to LCA, a statistical review of the experimental data provided a general overview on the variation in the growth performance traits between the two lines. The mean growth performance traits in the two lines were compared with a Student’s *t* test (Table 2) as well as the trait predictions from InraPorc^®^. The FCR differed significantly between the lines (−130 g/kg gain for LRFI compared to HRFI pigs; *P* < 0.001), as did the ADFI (*P* < 0.0001) and RFI (*P* < 0.01). The lines also differed in their ADG (*P* < 0.05), age at slaughter (*P* < 0.05), fattening duration (*P* < 0.05), but not in BW at slaughter (*P* = 0.43). The two lines had similar protein content at slaughter (*P* = 0.32), but not lipid content, back fat thickness and LMP (*P* < 0.0001), leading to a difference in the BP/BL ratio (*P* < 0.0001).

**Table 2:** Growth performance traits and InraPorc^®^ estimations of body composition of pigs in low residual feed intake (LRFI) and high residual feed intake (HRFI) lines

	Mean LRFI	Mean HRFI	Mean differences (%)	SD LRFI	SD HRFI	*P* ^1^
Traits records						
BW birth (kg)	1.50	1.53	1.98	0.20	0.33	0.63
BW weaning (kg)	8.51	9.12	6.92	1.18	1.22	0.007
BW initial fattening (kg)	28.7	29.9	4.09	4.06	4.70	0.14
ADG fattening (kg/day)	0.80	0.83	3.68	0.080	0.071	0.047
ADFI fattening (kg/day)	1.97	2.15	8.73	0.21	0.19	<0.0001
FI fattening (kg)	214.3	225.5	5.09	18.3	28.1	0.011
FCR fattening (kg/kg gain)	2.45	2.58	5.16	0.16	0.18	<0.0001
RFI (g/day)	−36.1	35.1	197.1	130.8	104.8	<0.01
ECR fattening (MJ/kg gain)	23.78	25.03	5.45	1.63	1.77	<0.0001
Fattening duration (days)	109.6	104.9	4.38	12.00	9.34	0.02
Age at slaughter (days)	181.1	177.0	2.28	10.00	7.44	0.011
BW slaughter (kg)	116.3	117.4	0.94	7.04	8.30	0.43
InraPorc^®^ estimations						
PD fattening (g/day)	133.0	136.9	2.88	13.9	15.4	0.38
Carcass weight (kg)	91.9	92.7	0.86	5.56	6.55	0.43
Lipid weight at slaughter (kg)	22.4	25.7	13.72	3.28	4.11	<0.0001
BFT slaughter (mm)	15.3	16.5	7.54	1.20	1.49	<0.0001
Protein weight at slaughter (kg)	18.6	18.4	1.08	1.31	1.34	0.32
LMP (%)	60.9	58.8	3.50	2.00	2.01	<0.0001
LMC (kg)	55.9	54.5	2.53	3.93	3.72	0.042
BP/BL at slaughter	0.85	0.73	15.18	0.13	0.13	<0.0001

BW = body weight; ADG = average daily gain; ADFI = average daily feed intake; FI = total feed intake; FCR = feed conversion ratio; RFI = residual feed intake; ECR = energy conversion ratio; PD = protein deposition; BFT = back fat thickness; LMP = lean meat percentage; LMC = Lean meat content; BP/BL = ratio of body protein weight/ Body lipid weight at slaughter.

1 *P* were calculated via a *t* test on the line effect.

### Individual life cycle assessment on the low and high residual feed intake lines

**Figure 2:**
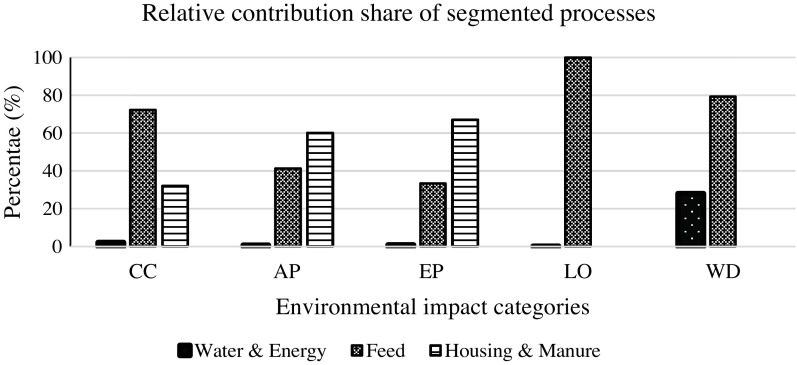
Relative contribution of the segmented pig farming processes within the system boundary of life cycle assessment (LCA), in the five impact categories. Feed ingredients are clustered as 1. feed; 2. emissions and excretion during housing, manure storage and spreading are clustered as housing and manure; 3. On-farm consumption of water and energy are clustered as on-farm water and energy. CC = climate change; AP = acidification potential; EP = freshwater eutrophication potential; LO = land occupation; WD = water depletion.

The five impact categories were calculated for 116 pigs through individual LCA. The outcomes of individual LCA on the LRFI and HRFI lines in the five impact categories are summarised in Table 3. The values in all impact categories were lower in the LRFI line than in the HRFI pigs (*P* < 0.0001): CC (2.60 *v*. 2.77 kg CO_2_-eq), AP (44.5 *v*. 48.1 gr SO_2_-eq), EP (3.35 *v*. 3.63 g P-eq), LO (4.19 *v*. 4.45 m^2^a) and WD (0.044 *v*. 0.047 m^3^). The minimum and the maximum differences between HRFI and LRFI were in LO (6.01%) and EP (8.02%), respectively, and the average difference for the five impact categories was 7%. To test the relative contributions of the different processes involved in the LCA, the impact categories were segmented into feed, housing and manure and on-farm water and energy (electricity, gas, etc.) use. Their percentage contribution to each segment is shown in Figure 2 for the two lines combined, as there were limited line differences. Feed had the maximum share in the impact categories of CC (72%), LO (100%) and WD (79%), whereas housing and manure had the biggest share in EP (66%) and AP (60%). On-farm water and energy had relevant impacts only in WD (28%).

**Table 3:** Five impact categories calculated per kg pig weight at farm gate by the life cycle assessment (LCA) model based on ReCiPe 2016 Midpoint (H) V1.13 method for low residual feed intake (LRFI) and high residual feed intake (HRFI) lines

Impact category	Unit	Mean HRFI	Mean LRFI	Difference (%)	SD LRFI	SD HRFI	*P* ^1^
Climate change	kg CO_2_ eq	2.77	2.60	6.33	0.12	0.11	<0.0001
Acidification	g SO_2_ eq	48.1	44.5	7.77	2.91	2.61	<0.0001
Eutrophication	g P eq	3.63	3.35	8.02	0.22	0.20	<0.0001
Land occupation	m^2^a	4.45	4.19	6.01	0.19	0.18	<0.0001
Water depletion	m^3^	0.047	0.044	6.59	0.0018	0.0017	<0.0001

P = phosphorous; m^2^a = area time; m^3^ = cubic meter;

1 *P* were calculated via a *t* test on the line effect.

The correlations between impact categories and performance traits, obtained from experimental data (ADG, FCR, ADFI and RFI) and traits simulated by InraPorc^®^ (BP/BL ratio, BFT, PD, BL, and BP), are reported in Table 4. Based on the 95% CI of the correlation estimations, no line differences were evident, except for BP with EP and AP, with a higher negative correlation in LRFI line. All impact categories were highly correlated to FCR, with values higher than 0.96 for both lines. All impact categories had moderate to high correlations with RFI (from 0.51 in HRFI pigs to 0.74 in LRFI pigs) and BP/BL ratio (values between −0.68 and −0.85). All impact categories are highly correlated to BFT, BP, BL and PD, with the absolute values higher than 0.48 for both lines except BP for HRFI line whose correlations had lower magnitude with AP and EP.

**Table 4:** Phenotypic correlations (95% CI) of five environmental impact categories with the recorded traits in the low residual feed intake (LRFI) and high residual feed intake (HRFI) pig lines

Trait	CC	AP	EP	LO	WD
LRFI line					
ADG fattening	−0.32 (−0.53; −0.07)	−0.35 (−0.56; −0.1)	−0.35 (−0.56; −0.11)	−0.31 (−0.52; −0.06)	−0.30 (−0.52; −0.05)
FCR fattening	0.97 (0.95; 0.98)	0.96 (0.94; 0.98)	0.96 (0.93; 0.98)	0.98 (0.96; 0.99)	0.97 (0.95; 0.98)
RFI (g/day)	0.73 (0.58; 0.83)	0.74 (0.6; 0.84)	0.75 (0.61; 0.84)	0.71 (0.56; 0.82)	0.71 (0.55; 0.82)
ADFI fattening	0.29 (0.03; 0.51)	0.26 (0.00; 0.48)	0.25 (0.00; 0.48)	0.30 (0.05; 0.52)	0.31 (0.06; 0.52)
BP/BL ratio	−0.68 (−0.80; −0.51)	−0.68 (−0.80; −0.51)	−0.68 (−0.79; −0.51)	−0.68 (−0.80; −0.51)	−0.68 (−0.80; −0.51)
BFT	0.58 (0.39; 0.73)	0.59 (0.39; 0.73)	0.58 (0.38; 0.73)	0.61 (0.42; 0.75)	0.60 (0.41; 0.74)
PD	−0.58 (−0.73; −0.38)	−0.61 (−0.75; −0.42)	−0.62 (−0.75; −0.43)	−0.57 (−0.72; −0.36)	−0.56 (−0.71; −0.35)
BL	0.58 (0.39; 0.73)	0.59 (0.39; 0.73)	0.58 (0.38; 0.73)	0.61 (0.42; 0.75)	0.60 (0.41; 0.74)
BP	−0.56 (−0.71; −0.36)	−0.55 (−0.7; −0.34)	−0.55 (−0.71; −0.34)	−0.51 (−0.68; −0.29)	−0.52 (−0.69; −0.31)
HRFI line					
ADG fattening	−0.47 (−0.65; −0.23)	−0.37 (−0.57; −0.12)	−0.36 (−0.57; −0.11)	−0.41 (−0.61; −0.17)	−0.44 (−0.63; −0.21)
FCR fattening	0.98 (0.97; 0.99)	0.99 (0.98; 0.99)	0.98 (0.97; 0.99)	0.99 (0.99;1.00)	0.98 (0.97; 0.99)
RFI (g/day)	0.51 (0.29; 0.68)	0.55 (0.34; 0.71)	0.55 (0.34; 0.71)	0.53 (0.31; 0.69)	0.51 (0.29; 0.68)
ADFI fattening	0.21 (−0.06; 0.44)	0.31 (0.06; 0.53)	0.32 (0.06; 0.53)	0.27 (0.01; 0.49)	0.23 (−0.03; 0.46)
BP/BL ratio	−0.74 (−0.84; −0.59)	−0.83 (−0.90; −0.72)	−0.83 (−0.9; −0.73)	−0.77 (−0.86; −0.64)	−0.74 (−0.84; −0.59)
BFT	0.48 (0.26; 0.66)	0.62 (0.42; 0.75)	0.62 (0.43; 0.76)	0.55 (0.34; 0.71)	0.51 (0.28; 0.68)
PD	−0.66 (−0.79; −0.48)	−0.59 (−0.73; −0.38)	−0.58 (−0.73; −0.38)	−0.61 (−0.75; −0.42)	−0.63 (−0.77; −0.45)
BL	0.48 (0.26; 0.66)	0.62 (0.42; 0.75)	0.62 (0.43; 0.76)	0.55 (0.34; 0.71)	0.51 (0.28; 0.68)
BP	−0.16 (−0.40; 0.11)	−0.03 (−0.29; 0.23)	−0.03 (−0.28; 0.24)	−0.07 (−0.32; 0.20)	−0.11 (−0.36; 0.15)

ADG = average daily gain; ADFI = average daily feed intake; FCR = feed conversion ratio; RFI = residual feed intake; Outcomes from InraPorc^®^: PD = protein deposition; BFT = back fat thickness; BP/BL = ratio of body protein weight/ Body lipid weight; Outcomes from life cycle assessment: CC = climate change; AP = acidification potential; EP = fresh water eutrophication potential; LO = land occupation; WD = water depletion.

### Uncertainty analysis

**Figure 3:**
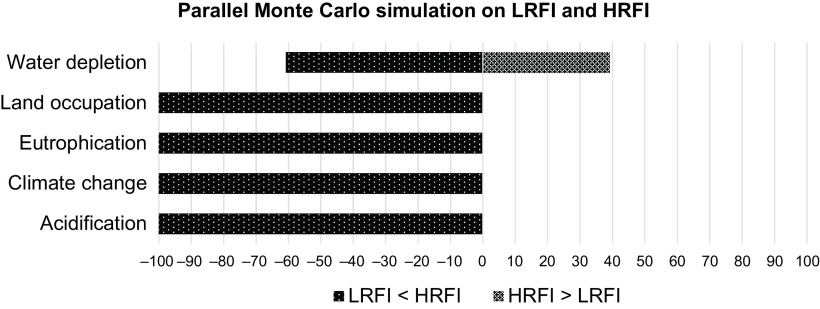
Life cycle assessment (LCA) applied to parallel Monte Carlo simulations for the high residual feed intake (HRFI) and low residual feed intake (LRFI) lines. The figure shows the percentage of scenarios from 1000 Monte Carlo simulations in which each line outperformed the other. Parallel Monte Carlo simulations use identical values from shared uncertainties to calculate environmental impacts. Therefore, the percentage difference in the results can be referred to as the difference between the lines. Positive values are associated with simulations in which the HRFI line has more favourable impacts than LRFI pigs, and negative values, the reverse.

A parallel Monte Carlo simulation study based on the mean values of the traits was run on both lines. The results are graphically represented in Figure 3 in five impact categories. In 100% of the simulations for CC, AP, EP and LO and 61% for WD, the LRFI line had less impacts than the HRFI line, indicating that the line differences are not sensitive to the uncertainty of the model parameters imbedded in SimaPro, except for WD.

### Sensitivity analysis

To perform the OAT sensitivity analysis, all incorporated production traits were kept constant, but the value of one trait was changed by ± 2 SD based on the distributions listed in Table 2. The focus traits BP, ADG, ADFI, PD, BL, FCR and BP/BL were changed OAT.

**Figure 4:**
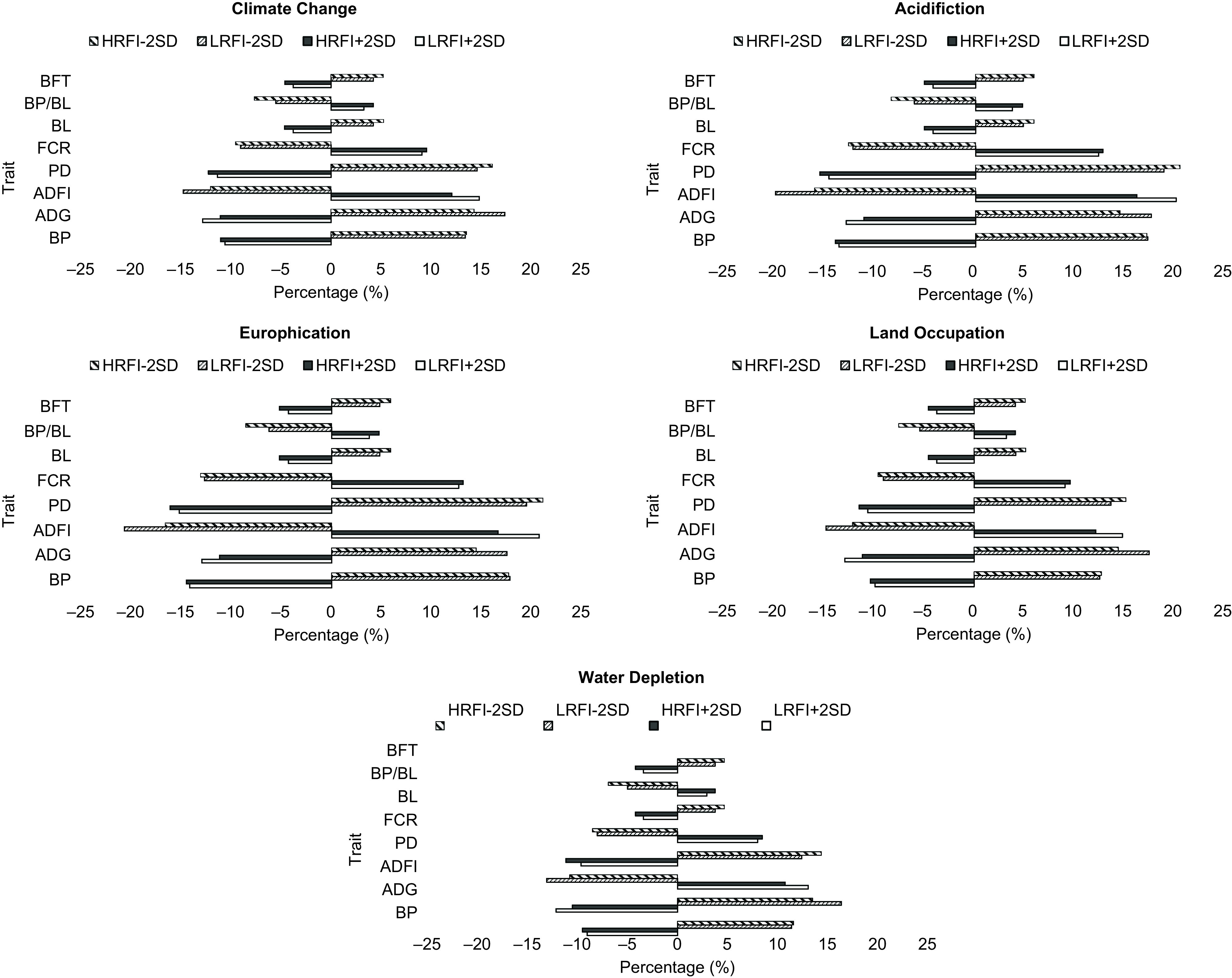
One-at-a-time sensitivity analysis based on the performance traits for the low residual feed intake (LRFI) and high residual feed intake (HRFI) pig lines. Percentage of changes in environmental impacts compared to the mean values due to changes in ± 2 SD in each trait. ADFI = average daily feed intake; ADG = average daily gain; BP = body protein at slaughter; BP/BL = ratio of body protein and body lipid at slaughter; PD = average daily protein deposition; BFT= back fat thickness; FCR = feed conversion ratio; BL = body lipid content at slaughter.

The percentage change in the environmental impact categories compared to the initial impact values due to the changes in any trait are presented in Figure 4. For all categories, the environmental impacts were sensitive to ADFI, ADG, FCR, BP and PD, which corresponded to more than 5% changes in the impacts compared to the initial values. The maximum and the minimum sensitivity for ADFI (+20.6% and −10.7%) were related to EP and WD, for ADG (+17.6% and −10.5%) to LO and WD, for FCR (+13% and −8%) to EP and WD, for BP (+17.7% and −9%) to EP and WD and for PD (+21% and −16%) both maximum and the minimum sensitivity were related to EP.

## Discussion

The aim of this study was to develop a model to evaluate the environmental impacts of selection for feed efficiency using comparative LCA and to apply the model to individual records of two divergent pig lines after five generations of selection for RFI. The FCR is correlated with RFI, and selection for reduced RFI has been shown to also reduce the FCR in these lines (Gilbert *et al.*, 2017). Lower FCR is generally due to lower feed intake, higher BW gain or both. Major differences in ADFI in the two lines and minor differences in ADG indicated that lower FCR in LRFI was mostly due to lower ADFI, which matches the objectives of selecting for RFI and agrees with earlier results in the same lines at that stage of the selection experiment (Gilbert *et al.*, 2007).

Studies have reported a negative (favourable) correlation between RFI and body leanness (e.g. Cai *et al.*, 2008). On the other hand, energy partitioning between PD and fat deposition can be modified by improving the feed efficiency (Noblet and van Milgen, 2004). If the general weight gain was little affected by selection, the InraPorc^®^ model showed that the protein to lipid ratio differed significantly between the lines, mainly due to the significant differences in lipid content at slaughter, meaning that selection for LRFI improved the protein to lipid ratio mainly through reduced LD and back fat thickness, in agreement with the hypothesis stated by Dekkers and Gilbert (2010) concerning the switch of more efficient pigs to a more oxidative metabolism.

Inferring from the differences between LRFI and HRFI feed intake, we hypothesised that the lines would have different environmental impacts. Indeed, the LRFI impacts were on average 7% lower than HRFI impacts in all categories, in agreement with the positive genetic correlation between FCR and RFI with excretion traits (N and P) reported by Saintilan *et al.* (2013) and Shirali *et al.* (2012) who used models at the level of the animal only to predict individual excretion of pigs.

Differences in the level of environmental impact categories between different LCA studies may be due to the differences in the methods, inventories, assumptions, emission factors and system boundaries. To guarantee consistency in the calculation model, LCA method, inventories and system boundary, when comparing the lines, we applied the same model to both. By changing the method to the CML-IA baseline V3.04 (Center of Environmental Science of Leiden University, http://cml.leiden.edu/software/data-cmlia.html) with the same inventories, the impact values decreased to 2.56 kg CO_2_-eq for LRFI and to 2.70 kg CO_2_-eq for HRFI, confirming the importance of the model for comparing impacts. Thus, although it may not be reasonable to compare the results of two different studies, one can reasonably compare their orders of magnitude and range. The values of the CC impact for LRFI and HRFI were in the same ranges as the values reported by Dourmad *et al.* (2014) (2.3 to 3.5 kg CO_2_-eq/kg LW) and de Vries and de Boer (2010) (2.3 to 5.0 kg CO_2_-eq/kg LW) for typical European production farms. The impacts of LRFI and HRFI on AP were also in the range of values reported by de Vries and de Boer (2010) (8 to 120 g SO_2_-eq/kg LW). The impact on EP for LRFI and HRFI differed from the impacts reported in the literature. These variations were due to the use of ReCiPe midpoint 2016, which accounts for the impact of freshwater EP based on P-eq rather than PO_4_-eq. When EP was calculated based on PO_4_-eq (according to the CML-IA baseline method) the values changed to 25 g PO_4_-eq for LRFI and 27 g PO_4_-eq for HRFI, which is in the same range of values reported by de Vries and de Boer (2010) (12 to 38 g PO_4_-eq/kg LW). The LO values were also in the range reported by de Vries and de Boer (2010) (4.2 to 6.9 m^2^/kg LW).

Clustering the different processes involved in the system boundary provided further insights into the relative contributions of each segment to the impact categories, with limited differences between lines. The relative importance of feed and manure were in accordance with the results published by Garcia-Launay *et al.* (2014). The higher feed contribution to three impact categories of CC, LO and WD is certainly the main driver of the higher environmental impacts of HRFI compared to LRFI. Moreover, as HRFI pigs consume more feed with limited difference in digestibility (Barea *et al.*, 2010; Montagne *et al.*, 2014), they excrete more nutrients and produce more manure because of the mass balance. Considering manure as organic fertiliser partly compensated for the higher environmental impacts of HRFI associated with higher excretion and emission rates. Relative contribution of the segmented process confirmed that improving feed efficiency and manure management presents the main opportunities for improvement in pig farming.

According to the average values of the traits, the RFI lines only marginally differ in BP and PD (*P* = 0.32). The PD plays a role in affecting the environmental impacts in two ways. On the one hand, BW is strongly dependent on protein accretion and LD (Noblet and Etienne, 1987), which could affect FCR. On the other hand, changes in protein content influence N retention and subsequent excretion. Excreted N is at the origin of the emissions of N gas as N_2_O and NH_3_ during animal housing, outdoor storage of manure and application of manure in the field. A change in body protein, on the one hand, alters FCR through a change in BW, and on the other hand, may – due to a domino effect – influence all downstream N-associated excretions and emissions. While all impact categories are moderately correlated to PD (−0.58), the marginal difference in the lines in BP suggests that selection for RFI would have only limited effects on PD and thus N excretion, which is one of the main sources of environmental impacts. However, the RFI correlations with impacts were of similar magnitude as PD, which could indicate that these two criteria would reduce the environment impacts partly via different levers. Thus, it could be inferred that selection for RFI could be combined with other criteria to target PD. In that respect, the close genetic correlation between FCR and lean meat growth rate (Clutter *et al.*, 2011) makes this trait a more promising criterion for environmental improvement, which from a practical perspective is interesting, as it has been for decades the main criterion used on pig farms to improve feed efficiency. The very high correlation between FCR and all impact categories confirmed FCR as a key trait to reduce the environmental burdens of pig production. However, selecting for FCR has major impacts on decreasing leanness, which might not be any more desirable for some commercial lines in the future. Our study shows that RFI would be a valid alternative to select for feed efficiency with positive environmental impacts.

The statistical analysis of the results of individual LCA, performed on all pigs, revealed that the lines are significantly different for the five categories of environmental impacts. The results of parallel Monte Carlo simulations confirmed these differences and showed that the line difference is not sensitive to the model parameter uncertainties. The OAT sensitivity analysis showed that the impact categories are highly sensitive to ADFI, PD, ADG and FCR and less sensitive to BFT, BL and BP/BL. On the other hand, the correlations between the impacts and the traits show that the impacts are highly correlated to FCR, BP/BL, BFT and BL. This discrepancy between the OAT results and the correlations obtained from individual LCA could be due to not considering the correlations between the traits in the OAT sensitivity analysis, as proposed by Ottosen *et al.* (2019). Consequently, further global sensitivity analyses accounting for trait dependencies should enable a more global understanding of the influence of genetic trait changes on the environmental impacts. Ultimately, this could be used to propose new selection indexes optimising the economic and environmental components jointly, as explored recently by Besson *et al.* (2020).

## Conclusion

The feed-efficiency concept arose from an economic incentive as the ratio of gain (pig weight gain) to cost (feed). To date, emissions associated with pig farming have not been accounted for in selection strategies, neither as a cost nor as an income. In the environmental context, P and N excretions, associated emissions and other fluxes emerge as main sources of the environmental burden of pig farming. Ignoring that economic drivers influence the main sources of environmental costs was pointed out, and we suggest that including environmentally optimised criteria could alleviate the environmental burden of pig production, while still satisfying economic requirements. Consequently, our study shows that more optimal selection criteria could emerge through restructuring the trait weights from an environmental point of view.

## Software and data repository resources

The model was not deposited in an official repository.

## Supplementary material

For supplementary material accompanying this paper visit https://doi.org/10.1017/S175173112000138X. click here to view supplementary material

## References

[ref1] Ali BM , de Mey Y , Bastiaansen JWM and Lansink AGJMO 2018 Effects of incorporating environmental cost and risk aversion on economic values of pig breeding goal traits. Journal of Animal Breeding and Genetics 135, 194–207. 29878493 10.1111/jbg.12331

[ref2] Barea R , Dubois S , Gilbert H , Sellier P , van Milgen J and Noblet J 2010 Energy utilization in pigs selected for high and low residual feed intake. Journal of Animal Science 88, 2062–2072. 20154162 10.2527/jas.2009-2395

[ref3] Besson M , Komen H , Rose G and Vandeputte M 2020 The genetic correlation between feed conversion ratio and growth rate affects the design of a breeding program for more sustainable fish production. Journal of Genetics Selection Evolution 52, 5. 10.1186/s12711-020-0524-0 PMC7006397 32033525

[ref4] Cai W , Casey DS and Dekkers JCM 2008 Selection response and genetic parameters for residual feed intake in Yorkshire swine. Journal of Animal Science 86, 287–298. 17998435 10.2527/jas.2007-0396

[ref5] Clutter, AC 2011 Genetics of performance traits. In The genetics of the pig, 2nd edition (ed. MF Rothschild and A Ruvinsky ), pp. 325–389. CAB International, Wallingford, UK.

[ref6] Dekkers JCM and Gilbert H 2010 Genetic and biological aspect of residual feed intake in pigs. In Proceedings of 9th World Congress on Genetics Applied to Livestock Production, 1 August 2010, Leipzing, Germany, p. 287.

[ref7] Dourmad JY , Ryschawy J , Trousson T , Bonneau M , Gonzàlez J , Houwers HWJ , Hviid M , Zimmer C , Nguyen TLT and Morgensen L 2014 Evaluating environmental impacts of contrasting pig farming systems with life cycle assessment. Animal: An International Journal of Animal Bioscience 8, 2027–2037. 25170767 10.1017/S1751731114002134

[ref8] Dourmad JY , Etienne M , Valancogne A , Dubois S , van Milgen J and Noblet J 2008 InraPorc®: A model and decision support tool for the nutrition of sows. Animal Feed Science and Technology 143, 372–386.

[ref9] Garcia-Launay F , van der Werf HMG , Nguyen TTH , Le Tutour L and Dourmad JY 2014 Evaluation of the environmental implications of the incorporation of feed-use amino acids in pig production using Life Cycle Assessment. Livestock Science 161, 158–175.

[ref10] Gilbert H , Bidanel JP , Billon Y , Lagant H , Guillouet P , Sellier P , Noblet J and Hermesch S 2012 Correlated responses in sow appetite, residual feed intake, body composition, and reproduction after divergent selection for residual feed intake in the growing pig. Journal of Animal Science 90, 1097–1108. 22100596 10.2527/jas.2011-4515

[ref11] Gilbert H , Bidanel JP , Gruand J , Caritez JC , Billon Y , Guillouet P , Lagant H , Noblet J and Sellier P 2007 Genetic parameters for residual feed intake in growing pigs, with emphasis on genetic relationships with carcass and meat quality traits. Journal of Animal Science 85, 3182–3188. 17785600 10.2527/jas.2006-590

[ref12] Gilbert H , Billon Y , Brossard L , Faure J , Gatellier P , Gondret F , Labussière E , Lebret B , Lefaucheur L , Le Floch N , Louveau I , Merlot E , Meunier-Salaün M-C , Montagne L , Mormede P , Renaudeau D , Riquet J , Rogel-Gaillard C , van Milgen J , Vincent A and Noblet J 2017 Review: divergent selection for residual feed intake in the growing pig. Animal: An International Journal of Animal Bioscience 11, 1427–1439. 28118862 10.1017/S175173111600286X PMC5561440

[ref14] Huijbregts MAJ , Steinmann ZJN , Elshout PMF , Stam G , Verones F , Vieira MDM , Hollander A , Zijp M and van Zelm R 2016 ReCiPe a harmonized life cycle impact assessment method at midpoint and enpoint level. Report 1: characterization.

[ref13] IFIP 2014 La consommation d’eau en élevage de porcs et Les consommations énergétiques dans les bâtiments porcins by IFIP-Institut de la Filière porcine. Retrieved from https://www.ifip.asso.fr/sites/default/files/pdf-documentations/abreuvement-elevages-porc-ifip.pdf https://bio-e-co.fr/wp-content/uploads/2018/11/consommation_energie_elevage_porcs.pdf

[ref15] Intergovernmental Panel on Climate Change (IPCC) 2006. Guidelines for national greenhouse gas inventories, volume 4, IGES, Japan.

[ref16] Ivanov OL , Honfi D , Santandrea F and Stripple H 2019 Consideration of uncertainties in LCA for infrastructure using probabilistic methods. Structure and Infrastructure Engineering 15, 1–14.

[ref17] Kenny DA , Fitzsimons C , Waters SM and McGee M 2018 Invited review: improving feed efficiency of beef cattle – the current state of the art and future challenges. Animal: An International Journal of Animal Bioscience 12, 1815–1826. 29779496 10.1017/S1751731118000976

[ref18] Mackenzie SG , Leinonen I , Ferguson N and Kyriazakis I 2015 Accounting for uncertainty in the quantification of the environmental impacts of Canadian pig farming systems. Journal of Animal Science 93, 3130–3143. 26115299 10.2527/jas.2014-8403

[ref19] McAuliffe GA , Chapman DV and Sage CL 2016 A thematic review of life cycle assessment (LCA) applied to pig production. Environmental Impact Assessment Review 56, 12–22.

[ref20] McAuliffe GA , Takahashi T , Mogensen L , Hermansen JE , Sage CL , Chapman DV and Lee MRF 2017 Environmental trade-offs of pig production systems under varied operational efficiencies. Journal of Cleaner Production 165, 1163–1173. 29104375 10.1016/j.jclepro.2017.07.191 PMC5589118

[ref21] van Milgen J , Valancogne A , Dubois S , Dourmad J-Y , Sève B and Noblet J 2008 InraPorc®: A model and decision support tool for the nutrition of growing pigs. Animal Feed Science and Technology 143, 387–405.

[ref22] Montagne L , Loisel F , Le Naou T , Gondret F , Gilbert H and Le Gall M 2014 Difference in short-term responses to a high-fiber diet in pigs divergently selected for residual feed intake. Journal of Animal Science 92, 1512–1523. 24496835 10.2527/jas.2013-6623

[ref23] Nemecek T , Heil A , Huguenin-Elie O , Meier S , Erzinger S and Blaser S 2004 Life cycle inventories of agricultural production systems. Final Report Ecoinvent 15, 145–146.

[ref24] Nguyen TLT , Hermansen JE and Mogensen L 2010 Fossil energy and GHG saving potentials of pig farming in the EU. Energy Policy 38, 2561–2571.

[ref25] Nguyen TLT , Hermansen JE and Mogensen L 2011 Environmental assessment of Danish pork. Faculty of Agricultural Science, Aarhus University, Internal Report, p. 31 Retrieved from http://web.agrsci.dk/djfpublikation/djfpdf/ir_103_54761_indhold_internet.pdf

[ref26] Nguyen NH , McPhee CP and Wade CM 2005 Responses in residual feed intake in lines of Large White pigs selected for growth rate on restricted feeding (measured on *ad libitum* individual feeding). Journal of Animal Breeding and Genetics 122, 264–270. 16060494 10.1111/j.1439-0388.2005.00531.x

[ref27] Noblet J and Etienne M 1987 Body composition, metabolic rate and utilization of milk nutrients in suckling piglets. Reproduction, Nutrition, Development 27, 829–839. 10.1051/rnd:19870609 3659564

[ref28] Noblet J and van Milgen J 2004 Energy value of pig feeds: effect of pig body weight and energy evaluation system. Journal of Animal Science 82 (E-Suppl.), E229–E238. 10.2527/2004.8213_supplE229x 15471802

[ref29] Ottosen M , Stephen G , Mackenzie SG , Wallace M and Kyriazakis I 2019 A method to estimate the environmental impacts from genetic change in pig production systems. The International Journal of Life Cycle Assessment 25, 523–537.

[ref30] Quiniou N and Noblet J 2012 Effect of the dietary net energy concentration on feed intake and performance of growing-finishing pigs housed individually. Journal of Animal Science 90, 4362–4372. 22696619 10.2527/jas.2011-4004

[ref31] Rigolot C , Espagnol S , Pomar C and Dourmad J-Y 2010a. Modelling of manure production by pigs and NH3, N2O and CH4 emissions. Part I: animal excretion and enteric CH4, effect of feeding and performance. Animal: An International Journal of Animal Bioscience 4, 1401–1412. 22444660 10.1017/S1751731110000492

[ref32] Rigolot C , Espagnol S , Robin P , Hassouna M , Béline F , Paillat JM and Dourmad J-Y 2010b. Modelling of manure production by pigs and NH3, N2O and CH4 emissions. Part II: effect of animal housing, manure storage and treatment practices. Animal: An International Journal of Animal Bioscience 4, 1413–1424. 22444661 10.1017/S1751731110000509

[ref33] Saintilan R , Mérour I , Brossard L , Tribout T , Dourmad JY , Sellier P , Bidanel J , van Milgen J and Gilbert H 2013 Genetics of residual feed intake in growing pigs: relationships with production traits, and nitrogen and phosphorus excretion traits. Journal of Animal Science 91, 2542–2554. 23482579 10.2527/jas.2012-5687

[ref34] Saintilan R , Brossard L , Vautier B , Sellier P , Bidanel J , van Milgen J and Gilbert H 2015 Phenotypic and genetic relationships between growth and feed intake curves and feed efficiency and amino acid requirements in the growing pig. Journal of Animal Science 9, 18–27. 10.1017/S1751731114002171 25192352

[ref35] Sauvant D , Perez J-M and Tran G 2004. Tables of composition and nutritional value of feed materials. Wageningen Academic Publishers and INRAE, Paris, France.

[ref36] Shirali M , Doeschl-Wilson A , Knap PW , Duthie C , van Arendonk JAM , Roehe R and Kanis E 2012 Nitrogen excretion at different stages of growth and its association with production traits in growing pigs. Journal of Animal Science 90, 1756–1765. 22178856 10.2527/jas.2011-4547

[ref37] de Vries M and de Boer IJM 2010 Comparing environmental impacts for livestock products: a review of life cycle assessments. Livestock Science 128, 1–11.

[ref38] Wernet G , Bauer C , Steubing B , Reinhard J , Moreno-Ruiz E and Weidema B 2016 The ecoinvent database version 3 (part I): overview and methodology. The International Journal of Life Cycle Assessment 21, 1218–1230.

[ref39] Wilfart A , Espagnol S , Dauguet S , Tailleur A , Gac A , Garcia-Launay F 2016 ECOALIM: a dataset of environmental impacts of feed ingredients used in French animal production. PLoS ONE 11, e0167343. 27930682 10.1371/journal.pone.0167343 PMC5145169

[ref40] Young JM , Bergsma R , Knol EF , Patience JF and Dekkers JCM 2016 Effect of selection for residual feed intake during the grow/finish phase of production on sow reproductive performance and lactation efficiency. Journal of Animal Science 94, 4120–4132. 27898858 10.2527/jas.2015-0130

