# Supplementary material for: Evaluating environmental impacts of selection for residual feed intake in pigs
Source: Animal. 2020 Jun 22;14(12):2598–608. doi: 10.1017/S175173112000138X (PMC7645311; doi:10.1017/S175173112000138X)
Supplement: Supplementary file 1 [file S175173112000138Xsup001.docx]

Evaluating environmental impacts of selection for residual feed intake in pigs

T. Soleimani, H. Gilbert

*Animal* journal

To perform a sensitivity analysis, it is necessary to have a parametric model in which all parameters are mathematically interlinked. We used the following formulations for developing a parametric model to incorporate intended traits in our life cycle assessment (LCA) calculations.

eBW= 5.969*BP ^0.944^ + 0.854 * BL ^0.944^  (van Milgen et al., 2008)

Lean meat percentage= 72.58 – 43.49 * BL/ eBW (van Milgen et al., 2008)

N _Body_ = e^(-0.9892 – 0.0145 Lean%) * eBW^(0.7518 + 0.0044 Lean%) / 6.25

(Dourmad et al., 1992)

N _Intake_ = Feed _Intake_ * N _Feed_

N _Excreted_ = N _Intake_ – N _Retained_

P _Body_ (g)= 5.39*eBW (Rigolot et al., 2010a)

Ca _Body_ (g)= 8.56*eBW (Rigolot et al., 2010a)

K _Body_ (g)= - 0.0041*eBW^2^ + 2.68*eBW (Rigolot et al., 2010a)

Cu _Body_ (mg)= 1.1*eBW (Rigolot et al., 2010a)

Zn _Body_ (mg)= 20.6*eBW (Rigolot et al., 2010a)

N_2_0= 0.002*N _Excreted_ (Rigolot et al., 2010b)

N_2_= 5*N_2_0 (Rigolot et al., 2010b)

NH_3 Building_ (kg) = 17/14*0.24*N _Excreted_ (Rigolot et al., 2010b)

ResD= Feed _intake_ *residue feed

ECH_4 growing_ = ResD*670 J/g (Rigolot et al., 2010a)

CH_4_ _Emitted_= ECH_4_ / 56.65 MJ/kg (Rigolot et al., 2010a)

CH_4_ _Housing_ (kg) = VS*B_0_*MCF (Rigolot et al., 2010b)

OM _Faeces_ = Feed*OM_feed_ *(1 – dCOM) (Rigolot et al., 2010a)

dCOM _Grow_ = (0.744 + (14.69 DE – 0.50 NDF – 1.54 MM) / DM) / (OM / DM) (Rigolot et al., 2010a)

eBW = empty body weight ; BP = body protein ; L = body lipid; N _Body_ = nitrogen content of body; N _Intake_ = total uptaken nitrogen; N _Feed_ = notrogen content of 1kg feed; N _Excreted_ = total excreted nitrogen; N_Retained_ = nitrogen ratained in the body; OM = organic matter; MM = mineral mater; DM = dry matter; dCOM = feed organic matter digestibility coefficient; NDF = Neutral detergent fiber; B_0_ = maximum CH_4_ producing capacity; MCF = methane conversion factor; ResD = digsted fibre ingested.CH_4_ = methane; N = nitrogen; Ca = calcium; P = phosphorus; K = potassium; Cu = copper; Zn = zinc.
